# Uncertainty and Well-Being in Turkish Adults: Exploring the Role of Religiosity and Psychological Symptoms

**DOI:** 10.1007/s10943-025-02279-6

**Published:** 2025-02-27

**Authors:** Nuri Türk, Gökmen Arslan, Alican Kaya, Oğuzhan Yildirim

**Affiliations:** 1 Department of Guidance and Psychological Counselling, Siirt University, Siirt, Turkey; 2 Department of Guidance and Psychological Counseling, Mehmet Akif Ersoy University, Burdur, Turkey; 3 Department of Guidance and Psychological Counselling, Ağrı İbrahim Çeçen University, Ağrı, Turkey; 4 Department of Guidance and Psychological Counselling, Kahramanmaraş Sütçü İmam University, Kahramanmaraş, Turkey

**Keywords:** Religiosity, Life satisfaction, Intolerance of uncertainty, Depression, Anxiety, Stress

## Abstract

Religiosity offers individuals a sense of purpose and connection, which can lead to greater life satisfaction and improved psychological well-being and happiness. On the other hand, psychological challenges such as depression, anxiety, stress, and difficulty dealing with uncertainty can negatively affect life satisfaction. This study examined these dynamics using a hypothetical model, incorporating mediation and moderation analyses to explore the connections between intolerance of uncertainty, life satisfaction, religiosity, and psychological symptoms in a group of 565 participants (286 of whom were female). The findings revealed notable positive and negative relationships among these variables. Specifically, the study found that (i) religiosity and psychological symptoms both play a mediating role in the link between intolerance of uncertainty and life satisfaction, and (ii) religiosity serves as a moderator in this relationship. The moderation analysis showed that when religiosity levels are low, the negative effect of intolerance of uncertainty on life satisfaction is significantly stronger. In contrast, high levels of religiosity significantly weaken this negative relationship, indicating that religiosity acts as a buffer against the adverse impact of uncertainty on life satisfaction. These results underscore the potential value of religiosity in helping individuals cope with the harmful effects of uncertainty on their sense of life satisfaction. Additionally, lower levels of psychological symptoms and reduced intolerance of uncertainty were associated with higher life satisfaction, suggesting that religiosity may play a protective role in promoting overall well-being.

## Introduction

Life satisfaction, which represents happiness (Lyubomirsky et al., 2005), is a cognitive component of subjective well-being, and it refers to an individual’s cognitive judgments and evaluations of their life (Diener et al., 2002). High levels of life satisfaction are associated with many benefits (e.g., emotional, mental, social, and physical health). For example, individuals with high levels of life satisfaction have stronger disease resistance (Rosella et al., 2019), longer lives (Hudomiet et al., 2021), and better relationships (Amati et al., 2018). They also experience higher job satisfaction (Newman et al., 2015), self-confidence (Lyubomirsky et al., 2005), academic performance (Antaramian, 2017), fewer mental problems (Fergusson et al., 2015), and lower health problems (Hu et al., 2016). Therefore, understanding the factors associated with life satisfaction is crucial for developing intervention strategies to promote mental health and well-being (Tanhan, 2020; Tanhan et al., 2021). The current study aimed to explore the mediating and moderating role of religiosity and psychological symptoms in the association between intolerance of uncertainty and life satisfaction among Turkish adults.

### Intolerance of Uncertainty and Life Satisfaction

The intolerance of uncertainty refers to the inability to cope with negative emotions triggered by uncertainty (Carleton, 2016). In addition, it is conceptualized as a combination of cognitive, emotional, and behavioral reactions to uncertainty (Freeston et al., 1994). Individuals with high intolerance of uncertainty are unhappier (Evli & Şimşek, 2022) and have lower levels of well-being (Kareem et al., 2022; Shanza et al., 2021; Uzun, 2024). Moreover, intolerance of uncertainty, which plays a critical role in mental health (Del Valle et al., 2020; Smith et al., 2020), increases mental distress (McCarty et al., 2022) and decreases life quality and satisfaction (Karataş & Tagay, 2021).

Intolerance of uncertainty is a critical problem for young people struggling to decide about their future (Akshaya Dinarajan & Manikandan, 2022; Chen & Zeng, 2021). Unemployment, insufficient opportunities, and career indecision seriously disturb these young people (Adu et al., 2020; Jackson & Tomlinson, 2020). Therefore, these problems lead to uncertainty about future expectations for college students (James et al., 2021). Other factors associated with uncertainty are young people’s interests, abilities, and perceptions. Thus, these uncertainties can negatively affect young people’s mental health (Tsai et al., 2017). However, perceived problems about the future among young people reduce life satisfaction (Parola & Marcionetti, 2022). On the other hand, young people who have made certain decisions for their future have higher life satisfaction (Arslan, 2022; Sánchez-Sandoval et al., 2022).

Intolerance of uncertainty also plays a predictive role in anxiety and depression (Yuniardi, 2018). It is critical in developing depression and anxiety symptoms among young people in cognitive behavioral therapy (CBT) practices (Kendall et al., 2020; Laposa et al., 2022; Talkovsky & Norton, 2018). The CBT approach is often evaluated in conjunction with other theories (Lee & Cho, 2021; Maskey et al., 2019). For example, Koszycki et al. (2014) reported that the effectiveness of CBT and spirituality-focused psychotherapy were compared, and it was concluded that both approaches were effective in reducing symptoms of anxiety and depression. Spirituality psychotherapy can be successfully integrated into a cognitive behavioral approach, and this emerging approach has been shown to reduce psychological symptoms (Worthington et al., 2011).

### Religiosity and Psychological Symptoms

Depression and anxiety are recognized as severe disorders all over the world. According to the World Health Organization, more than 300 million people worldwide suffer from depression (WHO, 2017). The rate of people diagnosed with depression yearly is over 10% (Mclean et al., 2011). Similarly, anxiety is one of the most common mental illnesses. Depression, anxiety, and stress are related (Moutinho et al., 2017). These psychological symptoms have serious adverse effects on mental health, such as suicide (Lew et al., 2019; Sher, 2020), burnout (Koutsimani et al., 2019), anorexia nervosa (Lian et al., 2017), alcohol use disorder (McHugh & Weiss, 2019), and sleep disturbances (Franzen & Buysse, 2022). Protective factors are critical for reducing the risk of these symptoms, and life satisfaction is one of them (Chang et al., 2019; Zhang et al., 2017). On the other hand, depression and anxiety can negatively affect life satisfaction (Lopes & Nihe, 2021; Maria-Ioanna & Patra, 2020). In addition, depression and anxiety reduce the quality of life (Kugbey et al., 2020).

Another protective factor against symptoms of depression, anxiety, and stress can be considered religiosity. Religious coping can reduce depression, anxiety, and stress (Chow et al., 2021; Korbman et al., 2022). A meta-analysis has revealed that religiosity reduces anxiety and depression (Forouhari et al., 2019). This result emphasizes the importance of religiosity and draws attention to its preventive role. Religiosity supports increased mental health and growth (Arslan, 2025; Amrai et al., 2011). Furthermore, religious individuals have higher levels of well-being (Abdel-Khalek & Tekke, 2019; Moreno & Cardemil, 2018) and life satisfaction (Salsman et al., 2005). Religious young individuals have higher happiness and love for life (Abdel-Khalek & Singh, 2019). Lim and Putnam (2010) found that religious individuals have healthy social relationships through religious practices. Religious practices improve psychological well-being, life satisfaction, and social skills (Arslan, 2021; Papadopoulos, 2020). In addition, religious individuals can effectively cope with difficulties (Butler-Barnes et al., 2018; Pargament et al., 2011).

### The Present Study

Previous successful studies have shown a link between intolerance of uncertainty and life satisfaction (Odaci et al., 2022; Yang et al., 2016), as well as the association between life satisfaction and both religiosity (Vang et al., 2019; Yeniaras & Akarsu, 2017) and psychological symptoms (Aziz & Tariq, 2019). Given the theoretical framework of the cognitive behavioral therapy approach (CBT) and the aforementioned research, examining life satisfaction in terms of intolerance of uncertainty, religiosity, and psychological symptoms and understanding appropriate precautions and strategies to increase well-being is critical. Therefore, the hypotheses for this study are based on the literature on life satisfaction, intolerance of uncertainty, religiosity, and psychological symptoms, which are as follows: (H_1_) Intolerance of uncertainty predicts life satisfaction. (H_2_) Religiosity mediates the relationship between intolerance of uncertainty and life satisfaction. (H_3_) Psychological symptoms mediate the relationship between intolerance of uncertainty and life satisfaction. (H_4_) Psychological symptoms and religiosity have a serial mediator effect on the relationship between intolerance of uncertainty and life satisfaction. (H_5_) Religiosity moderates the relationship between intolerance of uncertainty and life satisfaction.

## Method

### Participants

The current study sample consisted of 565 individuals (286 female) (see Table 1). The participants ranged from 18 to 67 years old, with a mean of 26.97 (SD ± 9.30). The majority of participants called themselves religious (506 (90%)). Moreover, 341 (60.4%) participants identified themselves as Turkish. Self-expressed socioeconomic levels (SESL) of participants were (149 (26.4%)) low, (357 (63.2%)) moderate, and (59 (10.4%)) high.

**Table 1:** Demographic characteristics of participants (*N* = 565)

Variables		*N*	Percentage (%)
Gender	Female	286	56.1
	Male	279	43.9
Participant numbers who identify as religious	I’m religious	506	89.6
	I’m not religious	59	10.4
Ethnic identity	Turkish	341	60.4
	Kurdish	175	31.0
	Arab	34	6.0
	Prefer not to answer	15	2.6
Self-reported socioeconomic levels	Low	149	26.4
	Moderate	357	63.2
	High	59	10.4
Religious sects	Hanafi	326	57.7
	Shafi’i	233	41.2
	Hanbali	4	0.7
	Maliki	2	0.4
Total		565	100

### Power Analysis

Power analysis was performed so that the relationships between the predictor and predicted variable determined within the scope of the study could be uncovered powerfully. The power analysis was carried out under specified conditions such as alpha level 0.05, beta level 0.20, effect size low level, and two-way hypothesis. The minimum number of participants for these conditions was determined to be 395. The analysis was repeated under the same conditions for 565 participants, and results showed that the power of the analysis was (1-*β*) = 0.918. According to this result, it was determined that the sample reached had enough power.

### Measures

#### Religiosity Scale (RS)

The 6-item RS (Zagumny et al., 2012; Turkish version: Ayten, 2014) was used to assess religiosity. Items (e.g., “*Religion influences all my activities in my life.*”) are rated on a five-point Likert scale from 1 (*It is not suitable for me at all*) to 5 (*It suits me completely*). The total score ranges between 6 and 30. The higher the score on the scale, the higher the participant’s perception of the influence of religion in personal life, their level of religious knowledge, and the importance of religious thoughts and activities for individuals. For the overall scale, Cronbach’s *α* was 0.91, and McDonald’s *ω* was 0.91 in the present study (Table 2).

**Table 2:** Religiosity scale items (Ayten, 2014)

Please tick (X) to indicate how appropriate the following statements are for you
My religion is something I often learn about
I strive to gain a deeper understanding of my religion
I value my religion because it provides answers to many questions about life’s meaning
My religious beliefs have a profound impact on how I view life
Religion influences all my activities in my life
It is very important for me to devote a certain amount of time during the day to prayer and religious reflection

Items are rated on a five-point Likert scale from 1 (*It is not suitable for me at all*) to 5 (*It suits me completely*)

#### Satisfaction with Life (SWLS)

The 5-item SWLS (Diener et al., 1985; Turkish version: Dağlı & Baysal, 2016) was used to assess meaning in life. Items (e.g., “*So far in life, I have the important things I want. I have been”* and “*I am satisfied with my life*”) are rated on a five-point Likert scale from 1 (*Completely disagree*) to 5 (*Completely agree*). The total score ranges between 5 and 25. The higher the score on the scale, the higher the satisfaction with life. For the overall scale, Cronbach’s *α* was 0.89, and McDonald’s *ω* was 0.90.

#### Depression, Anxiety, and Stress Scale (DASS-21)

The DASS-21 (Brown, 1997; Turkish version: Yılmaz et al., 2017) was used to evaluate participants’ depression, anxiety, and stress. The 21-item Turkish version of the scale is rated on a five-point Likert scale ranging from 0 (*Never*) to 4 (*Almost always*) (e.g., “*I cannot stand distractions from my work*” and “*I have difficulty breathing*”). The scale consists of three dimensions. A maximum score of 21 and a minimum score of 0 can be obtained from each dimension. The score varies between 0 and 21 in each dimension. High scores from these dimensions indicate an increase in depression, anxiety, and stress levels. For the overall scale, Cronbach’s alpha *α* = 0.94 and McDonald’s *ω* = 0.94.

#### Intolerance of Uncertainty Scale (IUS)

The 12-item IUS (Carleton et al., 2007; Turkish version: Sarıçam et al., 2014) was used to assess intolerance of uncertainty. Items (e.g., “*I cannot work well when I experience uncertainty*” and “*I cannot stand to be caught off guard*”) are rated on a five-point scale ranging from 1 (*It is not suitable for me at all*) to 5 (*It suits me completely*). The total score ranges between 12 and 60. The higher the score, the greater the intolerance of uncertainty. For the overall scale, Cronbach’s *α* was 0.94, and McDonald’s *ω* was 0.94.

### Procedure

After ethics committee approval was obtained, the online link was prepared. The link for the study was designed through *Google Forms*. The prepared link was sent via social media sites and online messaging platforms, including Facebook, Instagram, Twitter, and WhatsApp. Participants were informed about the duration and purpose of the study. Furthermore, they were also asked whether they voluntarily participated in the study. Only respondents who voluntarily participated in the study were allowed to proceed to answer. No fees were paid to participants. The individuals were reached via convenient sampling. The inclusion criterion was to be over 18 years of age. Those who did not want to participate voluntarily were excluded from the study. Initially, 593 participants completed the form. There were control questions in each scale battery. These questions were placed to measure the attention level of the participants, along with the warning, for example, “*Please mark 3 answers in this item*.” Eighteen participants failed to answer these control questions correctly. As a result of missing data and outlier analysis, a further ten individuals were excluded from the scope of the study. Finally, the research was carried out with 565 individuals (95.28% valid response rate). Participants were only allowed to complete the self-report surveys once in the study. In addition, participants were informed that they could stop responding to the questionnaires if, for any reason, they felt uncomfortable proceeding. Participants were also advised not to provide information about their personal information to protect their confidentiality. Approval was requested from all participants before they participated in the study. Ethics committee approval of this research was obtained from Siirt University (Reference number: 2486), and all research stages were carried out under the Declaration of Helsinki.

### Data Analysis

Prior to the analysis, the required assumptions for carrying out the analysis were tested. The prerequisites of parametric tests require the kurtosis and skewness values to be examined. No violation of assumption was found in the study data. Multicollinearity was also evaluated by using the study variables. An examination of the tolerance, variance inflation factor (VIF), and confidence interval (CI) values revealed that these values were within a range of acceptable thresholds. The cutoff values required to prevent multicollinearity problems are < 0.10 for tolerance value, < 10 for VIF value, and CI of 10–30 (Albayrak, 2005). Consequently, no multicollinearity problem was detected. Mahalanobis distance values were examined to determine the outliers. Ten outliers were determined in the dataset. The outliers were removed from the analysis. Overall, all analyses were performed with 565 samples. SPSS (version 26), Hayes’ (2018) PROCESS Macro (version 3), and G* Power 3.1.9.7 were used in all analyses.

## Results

Correlations between all variables are shown in Table 3 (intolerance of uncertainty, satisfaction with life, religiosity, and psychological symptoms). The result of person correlations showed that all variables were significantly (albeit moderately) related.

**Table 3:** Bivariate correlations and descriptive statistics among variables (N = 565)

	IOU	SWL	R	PS
Intolerance of uncertainty	–			
Satisfaction with life	− .49^**^	–		
Religiosity	− .23^**^	.41^**^	–	
Psychological symptoms	.45^**^	− .38^**^	− .20^**^	–
*Mean*	37.12	16.19	25.34	19.01
*Std. Deviation*	12.39	4.92	4.89	13.32
Skewness	− .55	− .06	− 1.3	.76
Kurtosis	− .26	− .46	1.6	.09

*IOU* Intolerance of uncertainty, *SWL* Satisfaction with life, *R* Religiosity, *PS* Psychological symptoms, *Std*. *Deviation* Standard deviation

^**^p < .001

### Serial Multiple Mediational Analyses

The results of the serial multiple mediational analyses are shown in Table 4. There was a direct effect of intolerance of uncertainty on satisfaction with life (*β* = 0.194, *p* < 0.001). When two mediators (religiosity and psychological symptoms) were included at the same time, the analysis results showed that the coefficient was still significant (*β* = 0.139, *p* < 0.001]. Intolerance of uncertainty was also found to positively predict psychological symptoms (*β* = 0.475, *p* < 0.001) and religiosity (*β* = *− *0.089, *p* < 0.001).

**Table 4:** Standardized indirect effect of intolerance of uncertainty on life satisfaction through religiosity and psychological symptoms

	Coeff.	95% CI	
		LL	UL
Intolerance ➔ Religiosity ➔Satisfaction with life	− .068^**^	− .1022	− .0432
Intolerance ➔ Symptoms ➔ Satisfaction with life	− .068^**^	− .1053	− .0364
Intolerance ➔ Religiosity ➔ Symptoms ➔Satisfaction with life	− .004^**^	− .0143	− .0035
Total effect	− .19^**^	− .2236	− .1663
Direct effect	− .14^**^	− .1698	− .1099
Total indirect effect	− .05^**^	− .0740	− .0373

*Coeff.* Coefficient, *CI* Confidence interval, *LL* Lower Limit, *UL* Upper Limit

^**^p < .001

There was also a significant indirect effect of intolerance of uncertainty on satisfaction with life via religiosity (indirect effect = *− *0.068, SE = 0.01, *95%* CI = [*− *0.1022, *− *0.0432]). Moreover, the indirect effect of intolerance of uncertainty on satisfaction with life via psychological symptoms was significant (indirect effect = *− *0.068, SE = 0.02, *95%* CI = [*− *0.1053, *− *0.0364]). In the last step of the analysis, the indirect effect of intolerance of uncertainty on satisfaction with life via religiosity and psychological symptoms was significant (indirect effect = *− *0.004, SE = 0.00, *95%* CI = [*− *0.0143, *− *0.0035]). Although the serial mediation effect is small, the confidence intervals excluded zero provide evidence that the mediation effect is significant (Hayes, 2015).

**Fig. 1:**
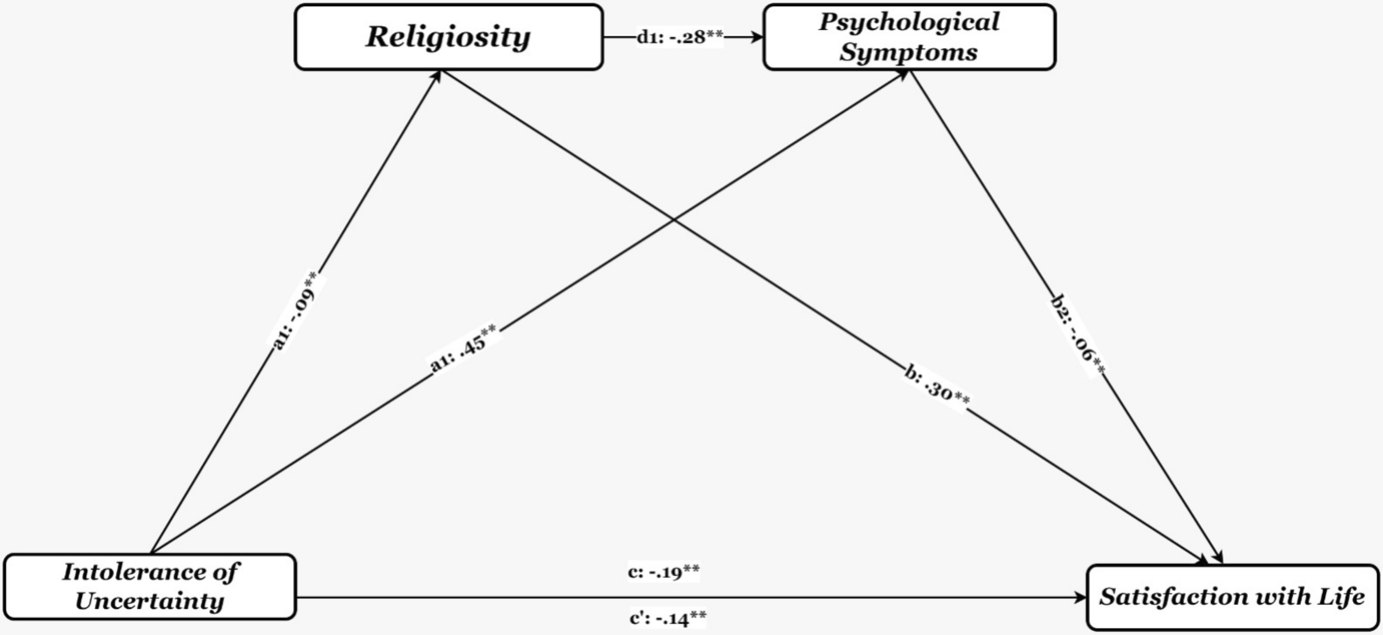
Results of the serial multiple mediational models

In conclusion, all hypotheses have been confirmed (see Table 4). The results confirmed that intolerance of uncertainty negatively predicted satisfaction with life. There were also indirect relationships between intolerance of uncertainty and satisfaction with life. The results also showed that religiosity and psychological symptoms partially mediated the relationship between intolerance of uncertainty and satisfaction with life (see Fig. 1).

### Moderated Mediation Analysis

The moderated mediation model was examined to determine whether happiness mediated the effect of intolerance of uncertainty on satisfaction with life. Religiosity moderated the mediating effect of satisfaction with life. To test H_4_, the PROCESS macro was used to conduct moderated mediation or conditional process analysis (Preacher & Hayes, 2004; Preacher & Hayes, 2008). A mediator (satisfaction with life) and a moderator (religiosity) were used in the model. The moderator simultaneously affects the effects of the bivariate relationship. As Table 4 shows, the results supported H_4_. Religiosity significantly affected the relationship between intolerance of uncertainty and satisfaction with life (*β* = 0.01, *p* < *0.0*01). So, the index of moderated mediation was significant (*β* = 0.01, *p* < *0.0*01; *95%* CI = [0.00, 0.01]).

**Fig. 2:**
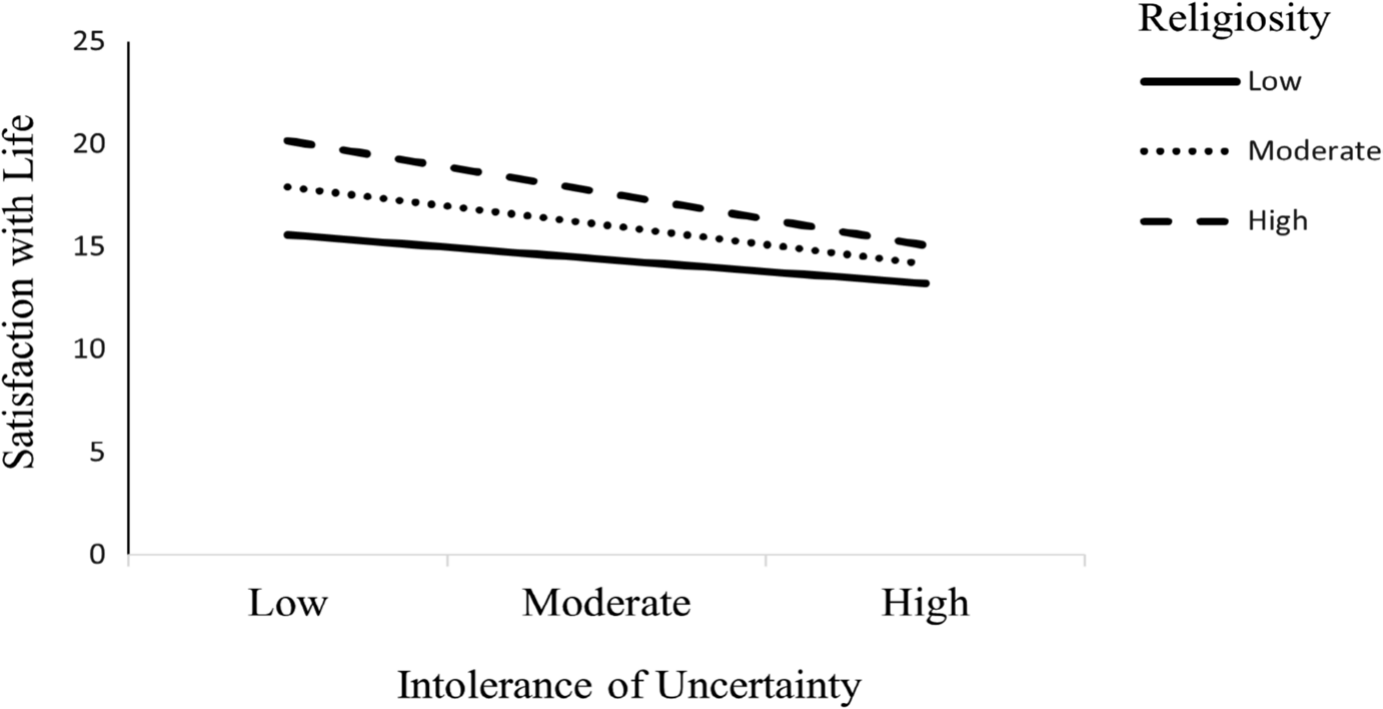
Results of the moderating effect of religiosity

In conclusion, the present study examined the conditional indirect effect of religiosity on psychological symptoms (via satisfaction with life) at three religiosity values: at 1 SD below, at the mean, and 1 SD above, respectively (see Fig. 2). The results supported H_5_. Table 5 shows the conditional indirect effect was significant at the low levels (β = *− *0.05, *95%* CI = [*− *0.01, 0.10]), moderate levels (β = 0.08, *95%* CI = [0.05, 0.12]), and high levels (β = 0.11, *95%* CI = [0.07, 0.17]). These results show that the indirect effect of intolerance of uncertainty (via satisfaction with life) on psychological symptoms is stronger at high levels compared to low levels of religiosity. For greater clarity, intolerance of uncertainty was more strongly linked to enhanced satisfaction with life when occurring in higher religiosity. In contrast, when religiosity occurs at low levels, intolerance of uncertainty is associated with lower life satisfaction. This finding shows that low religiosity negatively relates to the impact of intolerance of uncertainty on life satisfaction.

**Table 5:** Moderated mediational analyses

Antecedent	M (Satisfaction with life)			Y (Psychological symptoms)		
	Coeff.	SE	*p*	Coeff.	SE	*p*
*X* (Intolerance of uncertainty)	− .14	.08	> .05	− .37	.04	< .001
*M* (Satisfaction with life)				− .56	.11	< .001
*W* (Religiosity)	.77	.12	< .001			
X*W	.01	.00	< .001			
Constant	2.15	3.41	< .001	14.12	3.10	< .001
	*R* = .59; Δ*R*^2^ = .35			*R* = .48; ΔR^2^ = .23		
	*F* = 101.17; *p* < .001			*F* = 85.32; *p* < .001		

	Indirect effect	Boot SE	Boot LLCI	Boot ULCI
*Conditional indirect effect(s) of satisfaction with life on psychological symptoms*				
–1 *SD*	.05	.02	.01	.10
*M*	.08	.02	.05	.12
+ 1 *SD*	.11	.03	.07	.17

	Index	Boot SE	Boot LLCI	Boot ULCI
*Index of moderated mediation*				
Religiosity	− .0065	.0027	.0024	.0128

*Coeff*. Coefficient, *SE* Standard Error, *Boot* Bootstrapped, *CI* Confidence interval, *LL* Lower Limit, *UL* Upper Limit

## Discussion

This study examined the mediating role of religiosity and psychological symptoms (depression, anxiety, and stress) in the relationship between intolerance of uncertainty and life satisfaction. The present study’s finding shows that intolerance of uncertainty predicts life satisfaction (thereby confirming H_1_). The modern world has been adversely affected by difficult circumstances such as wars, migrations, and pandemics, contributing to increased uncertainty about the future (Kardaş, 2021).

Individuals who cannot tolerate uncertainty under challenging circumstances may feel unsatisfied with their lives (Karataş & Tagay, 2021). Similarly, studies reported that increased intolerance of uncertainty leads to decreased happiness (Khodarahimi et al., 2021) and well-being (Saricali et al., 2022). Moreover, some studies also reported that intolerance of uncertainty reduces life satisfaction (Lee et al., 2016; Turan, 2019). Therefore, the current study finding is consistent with the literature.

Another finding of the present study was that religiosity mediates the relationship between intolerance of uncertainty and life satisfaction (thereby confirming H_2_). This mediating role shows that intolerance of uncertainty has a low effect on religiosity. Countries like Turkey, which have high levels of religiosity and are developing, experience many uncertain situations, and people may become more resilient to uncertainty (Kumar et al., 2022). It may be possible that this resilience can reduce the effects of uncertainty on religiosity (Arslan & Wong, 2024). Besides, some studies have found that increasing intolerance of uncertainty also increases religiosity (Howell et al., 2019; Kumar & Voracek, 2022). These findings suggest that religiosity plays an important role in coping with uncertainty. In other words, individuals with difficulty tolerating uncertainty may accept religiosity as a means of coping. In addition, according to the mediating role of religiosity, religiosity increases life satisfaction.

These findings are supported by studies on different cultures and religious beliefs regarding the relationship between religiosity and life satisfaction (Aydogdu et al., 2021; Bigdeloo & Bozorgi, 2016; Lim & Putnam, 2010; Sholihin et al., 2022). However, some studies in the literature conclude that religiosity reduces life satisfaction (Yeniaras & Akarsu, 2017). These findings have been interpreted as the unattainability of religious goals, and not fulfilling the requirements besides believing in religion may reduce the satisfaction of individuals with life (Saritoprak et al., 2018).

There are also studies in which no significant relationship was found between religiosity and life satisfaction (Pöhls, 2021; Pöhls et al., 2020). A study conducted by Ten Kate et al. (2017) found that being Catholic positively affects life satisfaction, while being Protestant has no significant relationship with life satisfaction, and being Muslim has a negative association with life satisfaction. Considering the positive relationship between religiosity and life satisfaction observed among Muslim participants in the current study, it is understood that factors such as participants’ religion, country of residence, socioeconomic status, and social class are essential (Tay et al., 2014). Moreover, other studies report that living with religious people and being religious leads to greater happiness and life satisfaction (Begum & Osmany, 2016; Sujarwoto et al., 2018).

It has also been observed that being religious in a religious environment supports social acceptance and increases life satisfaction (Okulicz-Kozaryn, 2010; Ten Kate et al., 2017). However, a study found that although religious belief significantly affected life satisfaction, religious behaviors did not predict life satisfaction (Yoo, 2017). The religiosity scale used in the current study measures religious belief and fulfilling the necessary behaviors; hence, it does not match the results of Yoo’s (2017) research. Conversely, other studies have found that religious attendance increases life satisfaction (Berthold & Ruch, 2014; Lechner & Leopold, 2015). These findings suggest that religious beliefs can positively affect individuals’ life satisfaction when put into practice.

The present study also found that psychological symptoms mediate the relationship between intolerance of uncertainty and life satisfaction, thus confirming H_3_. Intolerance of uncertainty has been linked to increased levels of depression, anxiety, and stress among individuals (Dar, 2017), which in turn have significant negative relationships with life satisfaction (Bukhari & Saba, 2017; Lopes & Nihei, 2021). Similarly, this study found a significant positive relationship between intolerance of uncertainty and psychological symptoms and a significant negative relationship between psychological symptoms and life satisfaction. Furthermore, the study found that psychological symptoms predicted life satisfaction. This finding is consistent with previous studies showing that depression, anxiety, and stress predict life satisfaction (Koçak, 2021; Martins et al., 2022). These results suggest that psychological symptoms exacerbated by intolerance of uncertainty can play a crucial role in determining an individual’s level of satisfaction in life.

This study is unique in the life satisfaction literature because it demonstrates that psychological symptoms and religiosity have a serial mediating effect on the relationship between intolerance of uncertainty and life satisfaction, thus confirming H_4_. Specifically, the results indicate that psychological symptoms and religiosity partially mediate the relationship between intolerance of uncertainty and life satisfaction. Additionally, the study found that individuals with high levels of religiosity had lower levels of depression, anxiety, and stress, leading to increased life satisfaction. This finding is consistent with literature demonstrating the negative relationships between religiosity, depression, anxiety, and stress (Abdi et al., 2019; Koçak, 2021) and highlights the potential benefits of belief-based interventions in preventing these negative outcomes.

Depression, anxiety, and stress have been linked to decreased life satisfaction (Lopes & Nihei, 2021; Satici et al., 2021), and adverse situations such as the COVID-19 pandemic and economic crises have been shown to weaken psychological resilience and increase these negative symptoms (Arslan & Yıldırım, 2021; Batmaz & Meral, 2022; Batmaz et al., 2021, 2022; Doğrusever et al., 2022). Therefore, the study’s findings, conducted during the COVID-19 pandemic, support prior research.

The last finding of this study is that religiosity has a moderating role in the intolerance of uncertainty and life satisfaction (therefore confirming H_5_). According to this result, as the level of religiosity increases, the effect level of the path between intolerance of uncertainty and life satisfaction becomes stronger. Some studies have found that increasing religiosity decreases intolerance of uncertainty (Bardeen & Michel, 2017; Kasapoğlu, 2020). Contrary to these research findings, some other studies examining the relationship between religiosity and intolerance of uncertainty report that religiosity increases intolerance of uncertainty (Abramowitz & Buchholz, 2020; Rehman et al., 2021). In addition, this study found that religiosity and intolerance of uncertainty have a significant negative relationship. Similarly, as discussed above, although there are different results regarding the relationship between religiosity and life satisfaction in the literature, according to the results of this study, life satisfaction increases as religiosity increases. Besides, the study’s findings showed that life satisfaction increased as intolerance of uncertainty decreased. As a result, a high level of religiosity will decrease intolerance of uncertainty and increase life satisfaction, strengthening the effect level of the path between intolerance of uncertainty and life satisfaction.

## Limitations

There are some limitations in this study, as in all studies. The first limitation is that the study is cross-sectional. Mainly, causality cannot be determined in cross-sectional studies. However, different views suggest that mediation studies can be conducted cross-sectionally, longitudinally, and experimentally. However, it can be recommended that future studies be qualitatively, longitudinally, and experimentally diversified to obtain more accurate and comprehensive data. The study’s second limitation is the data collection via an online questionnaire. This situation may have affected the answers given by the survey participants (inability to pay attention, disregard).

The results of the current study should not be generalized to all of Turkey as the participants are individuals over 18. In addition, the sample was limited since it only included individuals aged 18 and older; therefore, people of different age groups, such as high school students and secondary school students, were not included. This issue may be particularly relevant regarding religiosity because religious beliefs, interpretations, and practices differ among provinces in the east and west of Turkey. Future studies should pay attention to differences to achieve more reliable results. Another issue that can be considered a limitation in the research is that the answers the participants gave are accepted as correct. However, reasons such as the desire for social acceptance and high self-perception may have guided the participants’ responses.

## Implications

Intolerance of uncertainty decreases religiosity and increases psychological symptoms. As a result, it reduces individuals’ satisfaction with life. The decrease in religiosity has a significant impact on this relationship by increasing the frequency of psychological symptoms and the level of satisfaction with life. The current study’s findings are important for educators, public and private healthcare institutions, and mental health professionals. In particular, this study highlights the importance of designing strategies to increase life satisfaction and policies that take into account the role of religiosity and psychological symptoms.

It may be concluded that there are various ways to increase life satisfaction, including increasing the level of religiosity and decreasing depression, anxiety, stress, and intolerance of uncertainty based on the study results. Moreover, religiosity can help reduce depression, anxiety, stress, and intolerance of uncertainty, making it a crucial factor in individuals’ psychological well-being. These findings contribute to the existing literature by providing new factors and models for promoting life satisfaction and positive mental health. To translate these findings into real-life practice, it may be appropriate for private and public health institutions to conduct risk screenings for depression, anxiety, stress, and intolerance of uncertainty.

Following these screenings, mental health professionals can develop practical training programs to help individuals at risk manage these conditions effectively. Additionally, researchers should pay attention to the context of religiosity and consider the distinction between belief and action attendance of religiosity. Educators and mental health professionals can help individuals develop beneficial thoughts and attitudes toward religiosity, which can improve their life satisfaction.

The researchers also can use Online Photovoice (OPV), one of the most recent and effective innovative qualitative research methods. The method allows the participants to express their own experiences with as little manipulation as possible, compared to traditional quantitative methods (Doyumğaç et al., 2021; Tanhan, 2020). Future researchers can adopt a recent OPV method for experiential activities to increase group and organizational synergy. Utilizing OPV in education, students can share pictures to increase their emotional awareness by making sense of their spiritual values and satisfaction with life. This approach can offer valuable practices for discovering new perspectives on the relationship between religiosity and psychological symptoms.

## Conclusion

Study findings showed that individuals with a high level of religiosity were more satisfied with life than those with less. As a result, life satisfaction can be increased with interventions in which religiosity is understood correctly in its context, and the ways of thinking it provides can be transferred to practice. Similarly, negative relationships were found between intolerance of uncertainty and life satisfaction. For this reason, it is seen that individuals who have learned to manage and accept uncertainty and have gained the ability to cope with it are more satisfied with life. In addition, when religiosity and depression, anxiety, and stress are included in the relationship between intolerance of uncertainty and life satisfaction, the effect of intolerance of uncertainty on life satisfaction decreases. This shows that religiosity and depression, anxiety, and stress have serial mediator roles between intolerance of uncertainty and life satisfaction. Finally, the fact that individuals have a high level of religiosity makes the effect of intolerance of uncertainty stronger on psychological symptoms through life satisfaction.

## Data Availability

The datasets generated during and analyzed during the current study are available from the corresponding author upon reasonable request.
